# Special Issue “Top-down Proteomics: In Memory of Dr Alfred Yergey”. Alfred Linwood Yergey, III, 17 September 1941–27 May 2018

**DOI:** 10.3390/proteomes8010001

**Published:** 2020-01-15

**Authors:** Jens R. Coorssen, Matthew P. Padula

**Affiliations:** 1 Departments of Health Sciences and Biological Sciences, Faculties of Applied Health Sciences and Mathematics & Science, Brock University, St. Catharines, ON L2S 3A1, Canada; 2 School of Life Sciences and Proteomics Core Facility, Faculty of Science, The University of Technology, Sydney, Ultimo 2007, Australia

Please see *J. Am. Soc. Mass Spectrom.* (2019) for a heartfelt obituary by P. Jane Gale and Stephanie Cologna, long-time associates and friends of Al [1].

Even after 19 months we remain in disbelief over the sudden passing of our wonderful friend. We have lost a beloved colleague, and science has lost a critical and passionate voice for focused, inter-disciplinary research, quantitative excellence and, to be blunt, a voice that routinely and meaningfully provided a well-considered reality-check on our ideas, directions, and approaches. For most of us that made Al a bastion of critical thinking and a model to aspire to. For a few, perhaps, they quietly knew he could call their bluff and gave him a wide and respectful berth.

The legacy of Al Yergey is the belief that if you are going to do something, make sure that you do it properly, rigorously and quantitatively—a sentiment best conveyed in “Proteomics Is Analytical Chemistry: Fitness-for-Purpose in the Application of Top-Down and Bottom-Up Analyses” [2]—which appealed to the field of proteomics to hold itself to the highest possible standards and respect the complementarity of all available approaches.

In this Special Issue, we have sought to further explore that ideal by publishing articles and reviews that demonstrate the state-of-the-art in top-down proteomics and the complementarity with bottom-up approaches. Emphasis is on the absolute need for analytical rigor and reproducibility. We thus seek to have a conversation about how top-down proteomics—indeed, proteomics as a whole—needs to progress to best serve analytical science, and the ever-growing role of proteomics as a cornerstone of modern research. Simply put, proteomes are composed of proteoforms (and the peptide sub-proteome) rather than simply amino acid sequences or open reading frames. We must be cognizant of this molecular reality and the way it impacts on our thinking in terms of dissecting molecular mechanisms and the identification of effective biomarkers and therapeutic targets. Do our most current and most widely touted approaches really capture, let alone address, this reality? How do we find a more collegial, collaborative, and complementary way forward? How do we respect a diversity of opinion, especially when it is supported by a convincing weight of evidence? At times in the process of collating this Special Issue, these ideals were not fulfilled showing that we still have a long journey ahead.

In the spirit of Al Yergey’s substantial legacy as both a researcher and genuine Renaissance man (his main hobbies were cycling, botanical drawing, brewing beer, and cooking—the last two, after all, being (bio)chemistry!), we hope this Special Issue can highlight more productive ways forward than the field has perhaps been entertaining for the last decade or so. This comes with the necessary recognition that no approach is “perfect;” however, by not recognizing the genuine pros and cons of the available analytical approaches—which has largely not been the case with the preponderance of dogma in the field—we cannot move effectively forward in the most productive, collaborative, and scientifically sound manner. A recent technology blog has named high-throughput and commercialization as critical objectives for the field of proteomics (i.e., to be ‘more like genomics’). While laudable and an important consideration, historically a rush to these objectives also tends to negatively impact analytical quality and the capacity to be quantitative. How long has it taken the field of genomics to achieve its current capacities? How complex is a given proteome relative to the corresponding genome? How do we balance that inherent complexity, the need for robust, quantitative, and “deep” proteome assessments, and the need to apply the best possible complementary analytical approaches to current biomedical and environmental research questions while still moving the technology forward? Is the field’s focus on instrumentation solutions overlooking the need to assess sample preparation techniques that could move proteomics further forward? Our suggestion is that, as a field, we begin to openly and transparently discuss the actual pros and cons of *current* methodologies in order to capitalize on the former and minimize the latter; this will undoubtedly also move the technology forward. Surprisingly, that may turn out to mean more than one type of technology as the pressing need to resolve intact proteoforms is becoming increasingly apparent.

Thus, in this Special Issue dedicated to Al Yergey, we find a diversity of research articles applying top-down techniques to analyse basic cellular processes in humans, medical treatments, and agriculture. The first (Furber et al. [3]) is likely to be Al Yergey’s last scientific contribution, investigating the use of thiol-labelling reagents to better understand proteoforms involved in the late steps in exocytosis and test some of the findings with functional assays. Al’s article is followed by three firmly focused on agriculture. Vincent et al. [4] have optimised top-down MS-based proteomics to study the cannabis proteome, a vastly understudied plant with a vast range of untapped medical applications whose use has recently been legislated in numerous jurisdictions. Functional foods also represent understudied proteomes and Tomazou et al. [5] investigated potential anti-microbial peptides from the milk and cheese of goats and sheep, while Gutierrez et al. [6] analysed saliva samples from pigs to determine early markers and mechanisms of disease-causing growth retardation. A Special Issue would not be complete without an article related to cancer and Dalzon et al. [7] have used 2D-PAGE to examine the promising anti-cancer effects of quinolone–copper complexes, validating their findings with functional biochemical assays.

In addition, the included review articles address questions in proteomics that we believe are poorly understood or misconstrued, and shine a light on these problems to initiate a conversation about how they might be most constructively addressed. In keeping with this ideal, Zhan et al. [8] reviewed a body of literature that cement the role of 2D-PAGE as an effective “pre-fractionation” method for the comprehensive characterisation of intact proteoforms by LC-MS/MS, providing significantly deeper proteome analyses than LC-MS/MS methods alone. This is followed in the same vein by O’Rourke et al. [9], who want to prompt a discussion about the conundrums related to how to properly normalise proteomics data to make biologically meaningful conclusions. The Special Issue closes with a review of top-down methods for studying skeletal muscle by Dowling et al. [10], focusing on the proteoforms of the contractile apparatus. We hope that the readers find these articles as interesting and compelling as we did during the editorial process.

In closing, we are reminded of the words of Al’s son and one of his daughters at his eulogy:

“[Al’s] sense of humor, and a sharp, some might say sarcastic, but realistic wit… [coupled with] …a keen, curious, and inquisitive mind.”

Karl Yergey

“Good food, good drinks, music, art, cycling, science, my Mom—all of these things he got to experience in his last day. To say he lived life fully would be an understatement. What can you do? You can live life fully… You can hug your loved ones. You can raise a glass of beer or wine tonight and toast Al.”

Wendy Meadows

Cheers, Al—to your so very important legacy and all we still have to learn from it and live-up to in doing the most critical science we possibly can. Your expectations of and challenges to the field are exactly what are needed for us to remain “realistic” and “inquisitive.”

We hope that Al would find this a fitting tribute to the scientist that we should all aspire to be.

Matt Padula and Jens Coorssen.
